# Homosynaptic plasticity induction causes heterosynaptic changes at the unstimulated neighbors in an induction pattern and location-specific manner

**DOI:** 10.3389/fncel.2023.1253446

**Published:** 2023-09-27

**Authors:** Ali Özgür Argunsah, Inbal Israely

**Affiliations:** ^1^Laboratory of Neuronal Circuit Assembly, Brain Research Institute (HiFo), University of Zurich, Zurich, Switzerland; ^2^Department of Molecular Biology and Genetics, Faculty of Engineering and Natural Sciences, Kadir Has University, Istanbul, Türkiye; ^3^Department of Physiology and Biophysics, University of Washington School of Medicine, Seattle, WA, United States

**Keywords:** dendritic spine, structural plasticity, synaptic plasticity, heterosynaptic plasticity, metaplasticity, naturalistic activity, neighboring spines LTP

## Abstract

Dendritic spines are highly dynamic structures whose structural and functional fluctuations depend on multiple factors. Changes in synaptic strength are not limited to synapses directly involved in specific activity patterns. Unstimulated clusters of neighboring spines in and around the site of stimulation can also undergo alterations in strength. Usually, when plasticity is induced at single dendritic spines with glutamate uncaging, neighboring spines do not show any significant structural fluctuations. Here, using two-photon imaging and glutamate uncaging at single dendritic spines of hippocampal pyramidal neurons, we show that structural modifications at unstimulated neighboring spines occur and are a function of the temporal pattern of the plasticity-inducing stimulus. Further, the relative location of the unstimulated neighbors within the local dendritic segment correlates with the extent of heterosynaptic plasticity that is observed. These findings indicate that naturalistic patterns of activity at single spines can shape plasticity at nearby clusters of synapses, and may play a role in priming local inputs for further modifications.

## 1. Introduction

Dendritic spines, which are the sites where postsynaptic elements of a synapse are located, are highly plastic and their structure is strongly coupled to their function. Depending on their biochemical state and the synaptic activation they are subjected to, they can express different types of plasticity such as early- or late-long-term potentiation (E-LTP or L-LTP, respectively) or long-term depression (LTD; Malenka and Bear, 2004). The activation of multiple synapses engages processes such as synaptic competition and co-operation (Fonseca et al., 2004; Fonseca, 2012) within the dendritic branch (Govindarajan et al., 2011), and these biochemical processes can spread to neighboring synapses (Engert and Bonhoeffer, 1997) triggering further compensatory modifications (Oh et al., 2015). Eventually, a slower but more global homeostatic mechanism normalizes synaptic weights and spine sizes throughout the neuron (Turrigiano and Nelson, 2004; Turrigiano, 2011; Hobbiss et al., 2018).

While multiple dendritic spines can be activated and studied using electrical stimulation, developments in the field of optics and caged compounds have allowed researchers to study their structure and function at the level of individual inputs (Pettit et al., 1997; Schiller et al., 1998; Matsuzaki et al., 2001; Kruijssen and Wierenga, 2019). Studies focusing on single spine plasticity and related signaling pathways often focused on inducing structural plasticity first, followed by electrical confirmation in order to understand how secondary biochemical interactions emerged following synaptic activation (Harvey et al., 2008; Govindarajan et al., 2011; Bosch et al., 2014; Chang et al., 2017).

A widely used paradigm for the induction of long-term potentiation (LTP) at single spines involves light-mediated delivery of 30 pulses of glutamate (4 ms-long pulse-width, at 0.5 Hz, lasting 60 s; Kruijssen and Wierenga, 2019), which we will refer to as 30-Reg (Reg: Regularly spaced; Harvey and Svoboda, 2007; Harvey et al., 2008; Govindarajan et al., 2011; Hobbiss et al., 2018; Argunsah and Israely, 2023). This 30-Reg paradigm has been shown to induce LTP at single dendritic spines and depending on the availability of plasticity-related proteins (PRP), this form of plasticity could lead to either E-LTP or L-LTP (Govindarajan et al., 2011; Argunsah and Israely, 2023). Many signaling pathways at single spines have been studied based on the structural LTP (sLTP) induced by this particular pattern (Harvey et al., 2008; Lee et al., 2009; Zhai et al., 2013; Bosch et al., 2014; Chang et al., 2017). Neighboring dendritic spines have often been used as unstimulated controls and have repeatedly been shown to not undergo drastic changes upon the expression of homosynaptic plasticity at a stimulated neighbor (Matsuzaki et al., 2004; Harvey and Svoboda, 2007; Govindarajan et al., 2011). Although these 30-Reg stimulation-based studies have shaped our understanding of single spine structural plasticity mechanisms, endogenous neuronal firing patterns are not composed of inter-spike intervals with that degree of regularity (Zador and Dobrunz, 1997; Paulsen and Sejnowski, 2000; Frerking et al., 2005; Argunsah and Israely, 2023).

We have recently shown that uncaging patterns that are modeled after a Poisson process (termed naturalistic stimulation patterns or NSPs) induce diverse homosynaptic plastic changes at stimulated single dendritic spines (Argunsah and Israely, 2023). Here, we further analyzed our data to see whether unstimulated neighboring spines of NSP-stimulated synapses showed similar plasticity compartmentalization as is the case for neighbors of spines stimulated with the 30-Reg pattern. We examined changes relative to location (local dendritic neighborhood) and time (post-induction longevity).

## 2. Materials and methods

Animal experiments were conducted in Champalimaud Centre for the Unknown according to the European Union regulations on animal care and use, with approval from the Portuguese Veterinary Authority (DGV). Cultured hippocampal slices were prepared from both male and female C57BL/6J (RRID:IMSR_JAX:000664) mice on postnatal days 7–10. Organotypic hippocampal slice cultures (Stoppini et al., 1991) were prepared in ice-cold ACSF containing 2.5 mM KCl, 26 mM NaHCO_3_, 1.15 mM NaH_2_PO_4_, 11 mM D-glucose, 238 mM sucrose, 1 mM CaCl_2_, and 5 mM MgCl_2_ and cultured on Millipore (Merck) membranes. The pH was adjusted to 7.3 and osmolarity to 300–310 mOsm. We have changed the culture media with a fresh one every 2–3 days. After 4–5 days of culturing slices, we sparsely transfected slices by a Helios gene gun (Bio-Rad) using gold beads (10 mg, 1.6 μm diameter, Bio-Rad) that were coated with 100 μg Afp-GFP plasmid DNA (Inouye et al., 1997) at 160–200 psi. The slices were maintained in an interface configuration with the following media: 1 × MEM (Invitrogen), 20% horse serum (Invitrogen), GlutaMAX 1 mM (Invitrogen), 27 mM D-glucose, 30 mM HEPES, 6 mM NaHCO_3_, 1 M CaCl_2_, 1 M MgSO_4_, 1.2% ascorbic acid, and 1 μg/ml insulin. The pH was adjusted to 7.3, and osmolarity was adjusted to 300–310 mOsm. All other chemicals were from Sigma unless otherwise indicated.

### 2.1. Patch-clamp electrophysiology

Hippocampal slice cultures were pre-incubated for 45–60 mins at 25°C and perfused continuously with ACSF. Voltage-clamp recordings were performed using 7–8 MOhm electrodes filled with a potassium-gluconate-based internal solution containing 136.5 mM K-gluconate, 9 mM NaCl, 17.5 mM KCl, 10 mM HEPES, and 0.2 mM EGTA. The pH was adjusted to 7.2 using KOH and osmolarity was adjusted to ~285 mOsm. Neurons were clamped at 65 mV. Recordings in which series resistance was higher than 25 MOhm were discarded, and stability was assessed throughout the experiment (±20%). Alexa 594 (Thermo Fisher) at 0.025 mM was added to the internal solution to visualize dendritic spines. uEPSC responses were evoked by glutamate uncaging. Signals were acquired using a multiclamp 700B amplifier (molecular devices), and data were digitized with a Digidata 1440 at 3 kHz. EPSC amplitudes were analyzed using custom software written in Matlab.

### 2.2. Two-photon imaging and glutamate uncaging

A Ti:Sapphire laser (Coherent Inc.) at a wavelength of 910 nm controlled by PrairieView software utilizing a galvanometer-based scanning system built around a BX61WI Olympus microscope was used for two-photon imaging and uncaging (Bruker, RRID:SCR_017142). The hippocampal organotypic slices were perfused with an oxygenated artificial cerebrospinal fluid (aCSF) solution containing 2 mM *CaCl*_2_, 1 mM *MgCl*_2_, 127 mM *NaCl*, 2.5 mM *KCl*, 25 mM *NaHCO*_3_, 1.25 *NaH*_2_*PO*_4_, and 25 mM D-glucose at a rate of 1.5 ml/min and a temperature of 38°C to maintain room temperature (25°C) in the chamber. Secondary or tertiary dendrites of CA1 pyramidal neurons were imaged using a water immersion objective (60X, 0.9 NA, Olympus) with a 10× digital zoom. Z-stacks were collected every 5 min starting 40 min before the induction of plasticity up to 4 h after the induction, with a 0.3 μm spacing between z-slices. XY pixel size was 0.0198 μm/pixel (19.8 × 19.8*nm*). Images were collected in 1024 × 1024 pixels resulting in a field of view of ~20 × 20 μm. The laser power and PMT gain settings for imaging were consistently maintained throughout the experiments. Glutamate uncaging has been performed using caged compound MNI-caged-L-glutamate (MNI-Glu, Tocris) after reconstituting it in aCSF without 1 mM *MgCl*_2_ or 2 mM *CaCl*_2_ to create a 10 mM stock solution. Individual aliquots were made to achieve a working concentration of 2.5 mM MNI-Glu in 3 ml volumes. *MgCl*_2_ and *CaCl*_2_ were added to the solution afterward. Each new batch of MNI-Glu was verified by delivering five 1ms-long pulses to single spines as uncaging evoked excitatory post-synaptic pulses (uEPSCs) were recorded through whole-cell patch clamp recordings. The laser power required to produce an uncaging evoked excitatory post-synaptic current (uEPSC) of comparable size was determined based on average spontaneous mEPSC amplitudes. To maintain stable MNI-Glu concentrations, one aliquot of uncaging aCSF was delivered in a closed re-circulation setting for each plasticity experiment. Uncaging patterns were applied to single spines by targeting the laser 0.5 μm away from the distal edge of the spine head. Uncaging was performed in the presence of 2.5 mM MNI-glutamate using 4ms-long laser pulses with a power of 30 mW (at 720 nm). The glutamate uncaging process was conducted without extracellular *Mg*^2+^ to enable the observation of long-lasting structural changes without interfering with plasticity-related proteins during whole-cell physiology. The dendritic segment containing the stimulated spine and unstimulated neighbors was imaged every 5 min for a baseline period of 20–30 min. The selected spine is stimulated using one of the uncaging patterns such as 30-Reg, NSP-Uni, NSP-Beg, NSP-End, and subsequently, the dendrite of interest was imaged every 5 min for up to 4 h in aCSF containing 0.5 μM TTX (Tocris).

### 2.3. Generation of naturalistic uncaging patterns

To generate spike sequences with inter-spike-interval distribution satisfying a Poisson process, we generated 10,000 patterns by applying the inverse transform sampling using the inverse function of the exponential distribution. Due to the probabilistic nature of the sampling, not all pulse trains had exactly 30 pulses. We picked the one that has exactly 30 pulses in 60 s to be able to have a fair comparison with the 30-Reg protocol. A total of 740 out of 10,000 sampled patterns had exactly 30 pulses. We picked three pulse trains out of these 740. In order to have some diversity, we chose one train that had 10 pulses every 20 s (NSP-Uni), another one that had 15 pulses in the first 20 s and another 15 in the last 20 (NSP-Beg), skewing more to the beginning of the stimulation, and the last one had 15 pulses in the first 40 s and 15 in the last 20 (NSP-End), skewing to the end of the stimulation. The details of the generation of these pulse trains can be found in Argunsah and Israely (2023) and the Matlab code to generate naturalistic uncaging patterns for induction of plasticity is publicly available.^1^

### 2.4. Quantification and statistical analysis

All analyses were performed using Matlab (MathWorks, RRID:SCR_001622). We quantified changes in spine volume using a Matlab-based toolbox that we developed called SpineS (Argunşah et al., 2022). Briefly, the dendritic spine head was segmented using a watershed-based algorithm (Erdil et al., 2012). Integrated fluorescence intensity inside the borders of the segment is used after normalizing with the median fluorescence intensity at the closest dendritic segment. The final normalization was performed on a per-spine basis as a percent of the average baseline value for that spine after normalizing with median dendrite fluorescence. We imaged a dendrite of interest every 5 min. Results are presented as mean ± SEM. Analysis in **Figure 3** shows the coefficient of variation at the normalized neighboring spine volumes over time which is a proxy for the spine motility. Analysis in **Figure 4** has been done by fitting a line using a robust linear regression model (the fitlm with “logistic” weight function in Matlab) at the normalized neighboring spine volumes at every time bin and plotting them as a function of distance from the stimulated spine. All statistical analyses were performed using custom code written in Matlab (MathWorks) and available from the corresponding author. The non-parametric Mann-Whitney *U*-test was used to compare spine volumes at any time bin versus baseline or different conditions. Time series were compared using repeated-measures ANOVA after creating a repeated-measures fir model using Matlab's *fitrm* function. Stars represent degrees of significance as follows: (*) = *p* < 0.05; (**) = *p* < 0.01; (#)= *p* < 0.0001). # sign is used instead (****) for convenience.

## 3. Results

### 3.1. Unstimulated neighbors of stimulated spines show similar, stimulation pattern independent, volume trends over time

**Figure 1:**
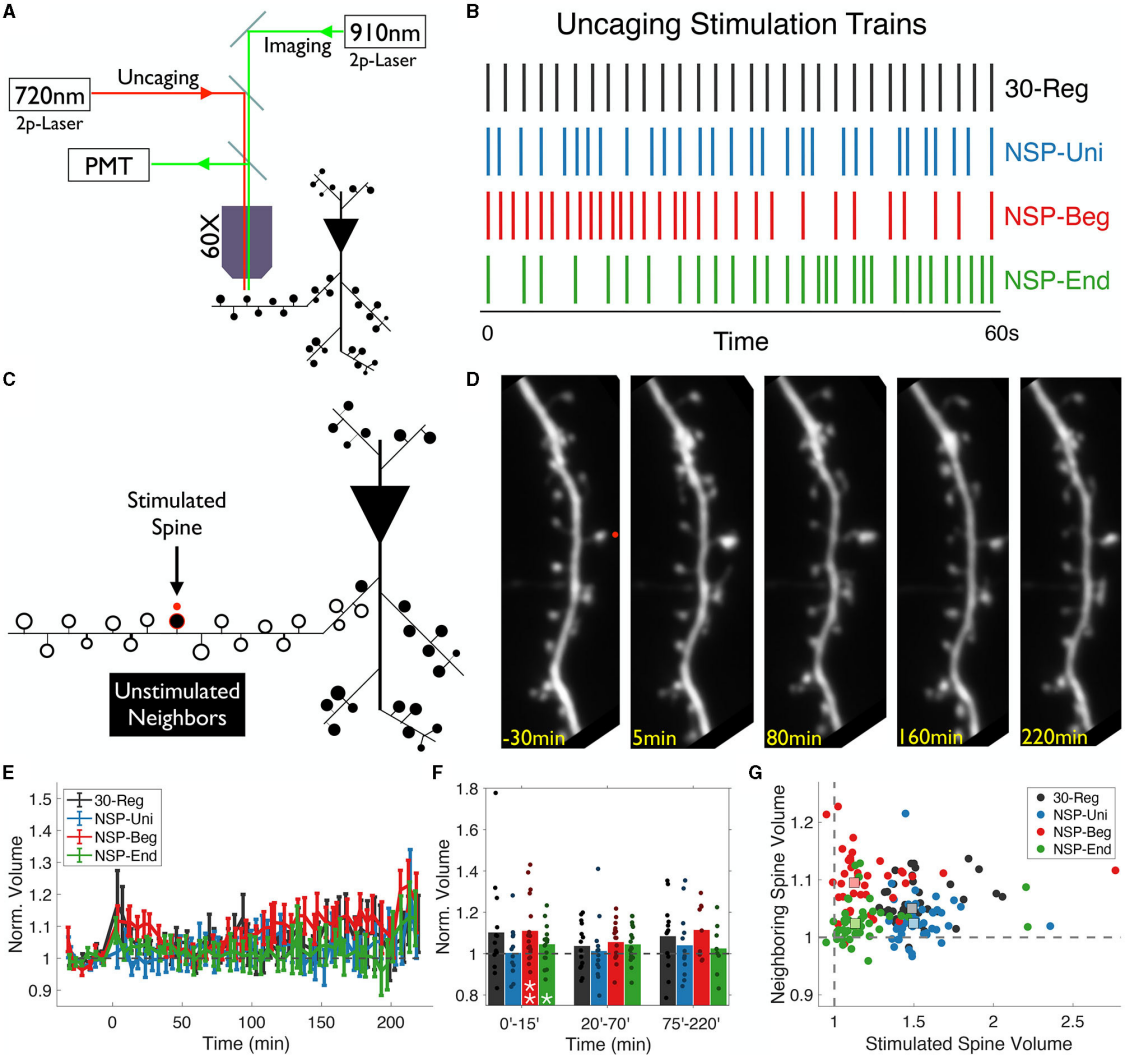
Single dendritic spine plasticity induction and visual tracking of the spines at the local dendritic segment. **(A)** Dendritic segments of GFP labeled hippocampal CA1 pyramidal neurons were imaged using a two-photon microscope with galvanometric scanning using a 910 nm laser with a 60X objective. Glutamate uncaging was delivered using a 720 nm laser. **(B)** Four different uncaging patterns were used to induce plasticity at dendritic spines. Bars represent uncaging pulses (4 ms width). All patterns have 30 pulses in 60 s. **(C)** Structural changes at the unstimulated neighboring spines around the stimulated spine at the local dendrite were analyzed. **(D)** Z-stacks of the dendritic branch of interest were imaged every 5 min before stimulation (40 min. baseline) and continued after for ~230 min post-stimulation. **(E)** Spine volumes are quantified for unstimulated neighboring spines for all four stimulation conditions and are represented as mean ± SEM (*P* = 0.427, repeated-measures ANOVA). **(F)** Only NSP-Beg and NSP-End induced sLTP at the neighbors of the stimulated spines in the first 15min post-induction. Black: 30-Reg (205 spines, 14 neurons, 11 animals); Blue: NSP-Uni (189 spines, 13 neurons, eight animals); Red: NSP-Beg (216 spines, 17 neurons, eight animals); Green: NSP-End (280 spines, 18 neurons, nine animals). **(G)** The average stimulated vs. unstimulated neighbor spine volumes for each condition. Dots represent average normalized volumes per time point. Boxes represent the median values of the averages. White stars represent statistical differences from baseline volume. Two-tailed non-parametric Mann-Whitney *U* was used for statistical comparison to the baseline. Repeated-measures ANOVA was used to compare conditions either fully in **(E)** or in time-series bins in **(F)**.

We used two-photon glutamate uncaging (720 nm) and imaging (910 nm) to induce and track the structural plasticity at single dendritic spines of CA1 pyramidal neurons (Figure 1A) using four different temporal uncaging patterns all containing 30 pulses in 60 s. Along with the classic 30-Reg pattern, we used three other patterns that are all sampled from the same Poisson distribution. We refer to these patterns as naturalistic stimulation patterns (NSP; Figure 1B). We chose these patterns since the endogenous activity in the hippocampus is well- modeled by this distribution (Rich et al., 2014). It is also consistent with the range of firing schemes observed in behaving animals in CA3 pyramidal neuron populations, which provide input to CA1 dendrites (Frerking et al., 2005).

We previously showed that temporal uncaging patterns derived from the same Poisson distribution with the same number of uncaging pulses as the regular train (30 pulses) induced plasticity at stimulated spines in a pattern-specific manner (Argunsah and Israely, 2023). Here, we expanded upon that idea by analyzing the volumes of the unstimulated neighbors surrounding the stimulated spines up to 15 μm in either direction along the dendrite (Figure 1C) and up to 4 h post-homosynaptic plasticity induction (Figure 1D). We have analyzed the normalized volumes of the unstimulated spines (Figure 1C) using the SpineS toolbox (Argunşah et al., 2022) before (baseline) and after (Figure 1D) the induction of single-spine plasticity (Figure 1F).

Previous studies, including from our laboratory, have shown that unstimulated neighbors of 30-Reg stimulated spines do not show significant structural fluctuations over time (Matsuzaki et al., 2004; Harvey and Svoboda, 2007; Govindarajan et al., 2011). Our initial analysis of the unstimulated spines confirmed this finding (Figures 1E, F, black). We first checked whether this finding holds for the unstimulated neighbors of NSP-Uni stimulated spines. We previously showed that while NSP-Uni induces long-lasting-sLTP up to 4 h post-induction like that induced by the 30-Reg pattern, NSP-Beg and NSP-End only induce short lived sLTP at stimulated spines that lasts at most 60 min (Argunsah and Israely, 2023; Figure 1G; black and blue vs. red and green). Our analysis of unstimulated neighbors of NSP induction showed that while neighbors of NSP-Uni stimulated spines did not show any structural plasticity, NSP-Beg and NSP-End induced slight but significant structural changes at the unstimulated neighbors compared to baseline in the first 15min post-induction (Δ*V*_*NSP*−*Beg*_ = 111 ± 4%, *Mean* ± *SEM, p* = 0.009; Δ*V*_*NSP*−*End*_ = 104 ± 2%, *Mean* ± *SEM, p* = 0.021, 0–15 min bin; Mann-Whitney *U*). When we compared all four patterns across several hours, no significant differences among conditions were observed [repeated-measures ANOVA (rmANOVA), 0–220 min post induction comparison, *p* = 0.4268, Figure 1E].

### 3.2. NSP-Beg induces heterosynaptic changes at the outer neighbors

It has been shown that functional properties of dendritic segments are region-specific (Yuste et al., 1994; Schiller et al., 1997; Larkum et al., 1999), yet in principle, they follow a gradient of synaptic integration along the proximal-distal axis. While distal synaptic inputs at a dendrite exhibit higher and broader input-output gain for temporal summation, proximal inputs to the same dendrite exhibit lower and narrower transfer functions (Destexhe, 2010). The activation of dendritic spines using glutamate uncaging in a centripetal or centrifugal direction leads to differential activation at the soma which is hypothesized to be due to the gradual activation of NMDARs along the dendrite (Branco et al., 2010; Branco and Häusser, 2011).

**Figure 2:**
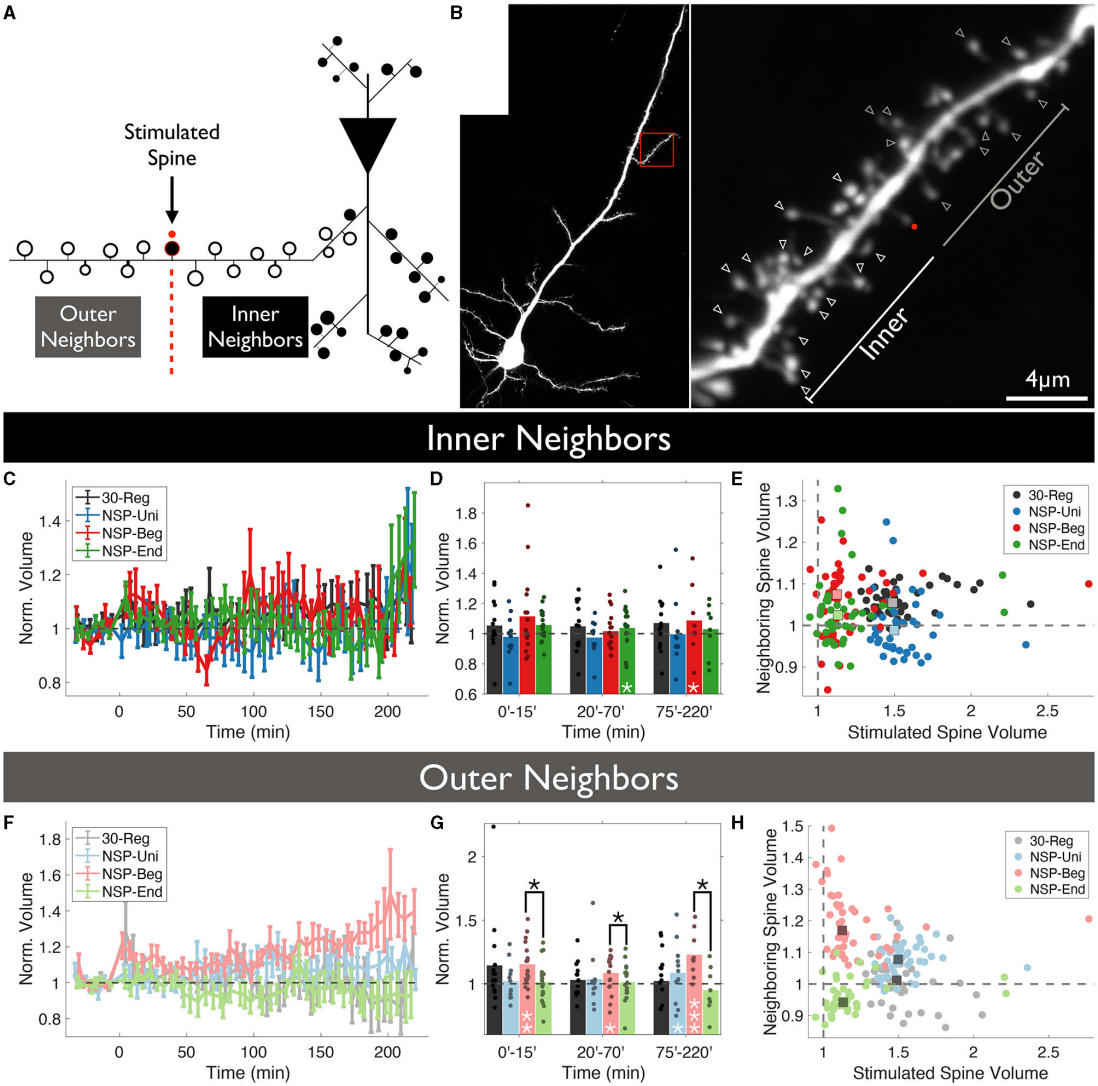
Location-dependent analysis of neighboring spine volumes. **(A)** Unstimulated spines around the stimulated spine are divided into two groups according to whether they locate between the stimulated spine and the main dendritic branch (*Inner*) or not (*Outer*). **(B)** Two-photon image of a CA1 pyramidal neuron in which a single spine undergoes plasticity induction. The red dot represents the uncaging point. The arrows represent the analyzed neighboring spines. Spines visible throughout the entire experiment were chosen to be analyzed. **(C)** Volume trends of *inner* neighbors (*P* = 0.47, RM-ANOVA). **(D)** Pair-wise comparison against baseline (white stars) and between conditions (black stars) for *inner* neighbors. **(E)** The average stimulated vs. unstimulated neighbor spine volumes for each condition. Dots represent average normalized volumes per time point. Boxes represent the median values of the averages. White stars represent statistical differences from baseline volume. Two-tailed non-parametric Mann-Whitney *U* was used for statistical comparison to the baseline. RM-ANOVA was used to compare conditions either fully in **(C)** or in time-series bins in **(D)**. **(F)** Volume trends of *outer* neighbors (*P* = 0.377, RM-ANOVA). **(G)** Pair-wise comparison against baseline (white stars) and between conditions (black stars) for *outer* neighbors. **(H)** The average stimulated vs. unstimulated neighbor spine volumes for each condition. Dots represent average normalized volumes per time point. Boxes represent the median values of the averages.

Following this idea, we wanted to test whether spines that are located between the stimulated spine and the primary dendrite show different structural dynamics compared to those that are further away from the stimulated spine near the distal end of the dendrite. We have named these neighboring spines as *Inner* and *Outer* neighbors, respectively (Figures 2A, B). Similar to previous results in which we did not divide spines according to their relative location to the stimulated spines, *inner* neighbors of the stimulated spines did not show any statistical differences in structural plasticity between the four conditions that were tested over time (rmANOVA, 0–220 min post-induction comparison, *p* = 0.4847, Figure 2C). However, we did see a significant sLTP at the neighbors of the NSP-Beg and NSP-End stimulated spines compared to the baseline at different time bins (Δ*V*_*NSP*−*Beg*_ = 109 ± 6%, *Mean* ± *SEM, p* = 0.049, at 75–220 min bin; Δ*V*_*NSP*−*End*_ = 104 ± 3%, *Mean* ± *SEM, p* = 0.024, at 20–70 min bin, Figure 2D). Quite interestingly, while outer neighbors of the stimulated spines did not show any statistical differences among the four conditions over time (rmANOVA, 0–220 min post-induction comparison, *p* = 0.3589, Figure 2F), neighbors of NSP-Beg and NSP-End stimulated spines had significant differences throughout the post-induction period (rmANOVA, 0–220 min post-induction comparison, *p* = 0.0150, Figures 2F–H red vs. green). This difference is most likely due to the significant deviation from the baseline volume of the neighbors of NSP-Beg stimulated spines (Δ*V*_*NSP*−*Beg*_ = 115 ± 4%, *Mean* ± *SEM, p* = 0.0180, 0–15 min bin; Δ*V*_*NSP*−*Beg*_ = 108 ± 4%, *Mean* ± *SEM, p* = 0.0177, 20–70 min bin; Δ*V*_*NSP*−*Beg*_ = 123 ± 3%, *Mean* ± *SEM, p* = 0.0295, 75–220 min bin; Mann-Whitney *U*).

### 3.3. Naturalistic patterns promote longer-lasting motility at the unstimulated neighbors

Dendritic spines are very plastic structures (Kasai et al., 2003), and particularly, spines of pyramidal neurons in the CA1 region of the hippocampus have a very high turnover rate (Attardo et al., 2015). Among other factors, synaptic activity is one of the main determinants of spine motility (Korkotian and Segal, 2001). Here, first we wanted to test whether different temporal patterns of synaptic inputs cause different levels of spine motility, and next, we wanted to check whether the motility is conditional to the relative directionality of plasticity at the stimulated spine.

**Figure 3:**
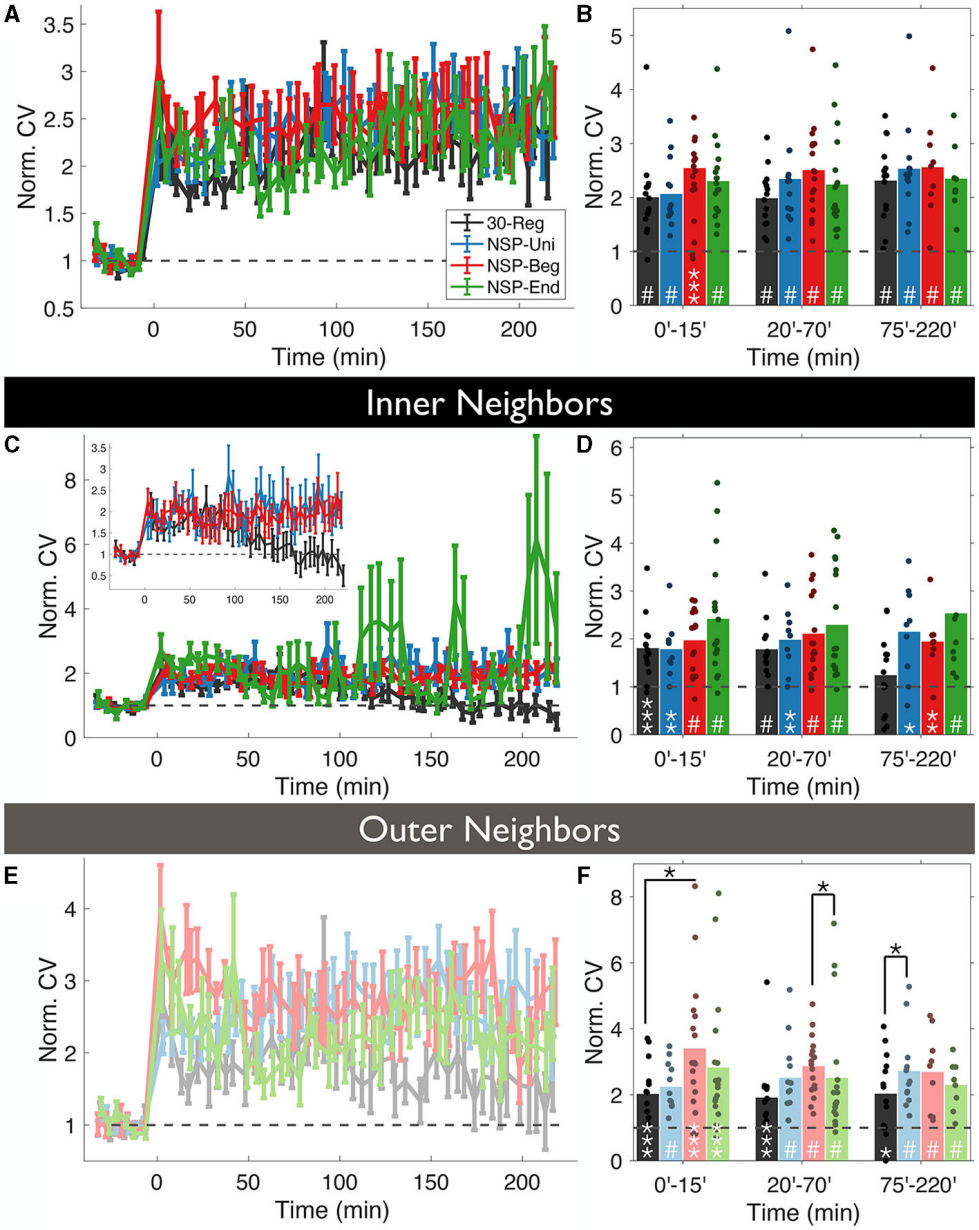
Volume variability of the unstimulated neighbors is conditional to the temporal pattern of the homosynaptic induction at the stimulated spine. **(A)** The coefficient of variation (CV) of normalized neighboring spines were calculated for each time bin using the formula $CV\left(t\right)=\frac{\sigma \left(t\right)}{\mu \left(t\right)}$ and normalized with the baseline CV. **(B)** CV fluctuates over time in an induction pattern-dependent manner. **(C, D)** Same as **(A, B)** but only for *inner* neighbors. Inset: Same as **(C)** without NSP-End. **(E, F)** Same as **(C, D)** but for *outer* neighbors. White stars represent statistical differences from the baseline. Two-tailed non-parametric Mann-Whitney *U* was used for statistical comparison to the baseline. Black starts represent statistical differences between conditions at a given time bin compared using RM-ANOVA.

First, we checked whether the uncaging pattern delivered at a stimulated spine has any effect on how much the volumes of the unstimulated neighbors will deviate around the mean (CV=$\frac{\sigma }{\mu }$, CV is coefficient of variation) over time. Overall, we did not see any significant differences among the four conditions (rmANOVA, 0–220 min post-induction comparison, *p* = 0.4472, Figure 3A), and every pattern caused highly significant deviations from baseline fluctuations (Figure 3B).

Divergences emerged when we divided unstimulated neighbors into *inner* and *outer* groups, according to dendritic location as in the previous analysis. Interestingly, the CV of *inner* unstimulated neighbors near 30-Reg stimulated spines returned to baseline levels approximately 2 h post-induction (Δ*V*_*NSP*−*Reg*_ = 125 ± 2%, *Mean* ± *SEM, p* = 0.3081, 75–220 min bin; Mann-Whitney *U*, Figures 3C, D), while *outer* ones did not (Δ*V*_*NSP*−*Reg*_ = 198 ± 3%, *Mean* ± *SEM, p* = 0.0163, 75–220 min bin; Mann-Whitney *U*, Figures 3E, F). This was not the case for any of the neighbors of NSP-stimulated spines (Figures 3C–F). Although the CV of *outer* neighbors near 30-Reg stimulated spines did not go back to baseline, they still exhibited significantly less CV than neighbors of NSP-Beg and NSP-Uni stimulated spines at different times (30-Reg vs. NSP-Reg: rmANOVA, 0–15 min post induction comparison, *p* = 0.0245; 30-Reg vs. NSP-Uni: rmANOVA, 75–220 min post induction comparison, *p* = 0.0430, Figure 3F).

### 3.4. NSP-Uni causes heterosynaptic plasticity gradients at the unstimulated neighbors around the stimulated spine along the local dendritic segment

**Figure 4:**
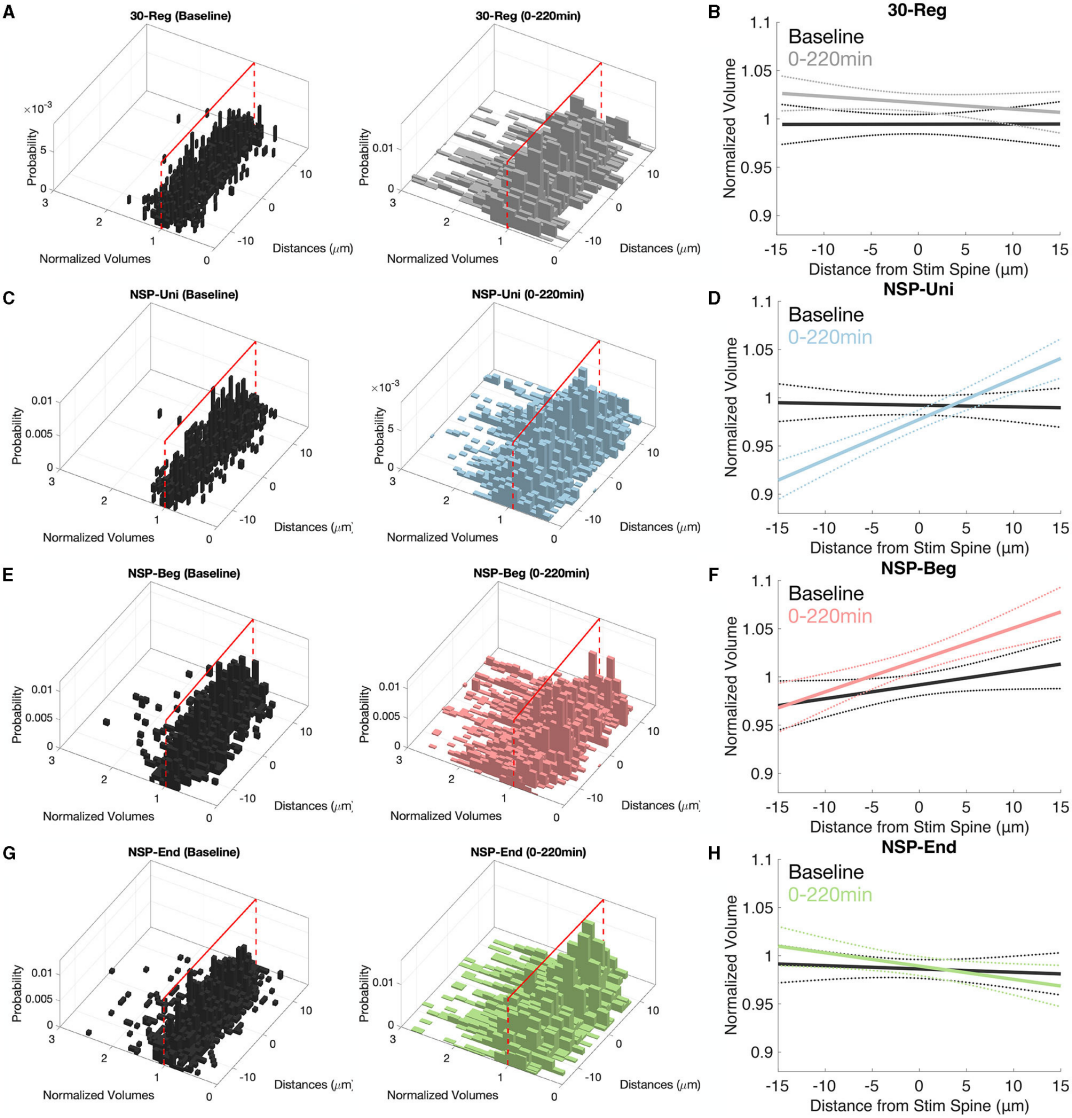
*Inner*–*Outer* neighbor plasticity gradients along pyramidal neuron dendrites. **(A, B)** There are no plasticity gradients around the 30-Reg stimulated spines. **(C, D)** While inner neighbors of NSP-Uni stimulated spines to show sLTD, this trend turns to sLTP at the outer neighbors. **(E, F)** Similar but marginal gradients as in **(D)** but for the neighbors of NSP-Beg stimulated spines. **(G, H)** There are no plasticity gradients around the NSP-End stimulated spines. Solid lines represent the average fit, and dotted lines represent 95% confidence intervals.

It has been shown that there are various gradients along the dendrites of pyramidal neurons such as the distribution of channels (Spruston, 2008), release probability, short-term facilitation (Grillo et al., 2018) and non-linear synaptic integration (Branco and Häusser, 2011). Here, we checked the possibility of heterosynaptic plasticity gradients at the unstimulated neighboring spines that could have been caused by the homosynaptic induction of plasticity at the stimulated spine. Neighbors of the 30-Reg stimulated spines did not show any plasticity gradients along their corresponding dendrites (Figures 4A, B, solid lines represent the mean of the fitted regression model, and dotted lines represent the 95% confidence interval). In stark contrast with the 30-Reg condition, there was a significant and stable increase in the neighboring spine volumes of the NSP-Uni stimulated spines in the direction of *inner*-neighbors → *outer*-neighbors (Figures 4C, D). While a similar gradient was caused by the NSP-Beg, the difference from the baseline gradient was marginal (Figures 4E, F). Similar to the 30-Reg condition, unstimulated neighbors of the NSP-End stimulated spines did not show any plasticity gradient (Figures 4G, H). The heteroplasticity gradient that we observe with NSP-Uni, but not with NSP-Beg or NSP-End, follows the successful induction of long term plasticity at the stimulated spine with only the NSP-Uni pattern as we previously described (Argunsah and Israely, 2023).

## 4. Discussion

Synaptic plasticity has often been studied at the site of induction using highly regular activation patterns either through the activation of multiple terminals by electrical means (Bear and Malenka, 1994) or at a single synapse level using glutamate uncaging (Kruijssen and Wierenga, 2019). Although regularly spaced stimuli have been the preferred choice of plasticity induction, *in-vivo* firing patterns are much more irregular and diverse (Connors and Gutnick, 1990). We have previously shown (Argunsah and Israely, 2023) that naturalistic patterns of activation can induce different types of plasticity at the stimulated dendritic spines in a pattern-dependent manner. Here, we further analyzed our data by focusing on the unstimulated neighboring spines to see whether the neighbors of NSP-stimulated spines showed any structural plasticity. It has repeatedly been shown that when plasticity is induced at single dendritic spines using glutamate uncaging with 30-Reg protocol, neighbors of these 30-Reg stimulated spines do not show any significant structural changes over time (Matsuzaki et al., 2004; Harvey and Svoboda, 2007) even if the experiment was performed under the influence of protein synthesis promoters such as forskolin (Govindarajan et al., 2011). However, when a group of spines were stimulated one by one using glutamate uncaging, unstimulated spines show heterosynaptic depression inside the cluster (Oh et al., 2015). Additionally, when a group of spines was activated individually but simultaneously at a local dendritic segment, bi-directional postsynaptic heterosynaptic plasticity was observed at unstimulated neighbors (30-Reg combined with voltage clamp at 0 mV) and the polarity of neighboring plasticity was a function of the distance between the center of the stimulated spines and the unstimulated neighbors (Tong et al., 2021). This led researchers, through computational modeling, to hypothesize that different activity patterns are likely to engage different endogenous dendritic mechanisms (Chater et al., 2022). Here, we confirmed this hypothesis by using two-photon glutamate uncaging and imaging and utilizing different stimulation uncaging patterns for the induction of homosynaptic plasticity. We have shown that the structural properties of unstimulated neighbors are conditional in combination with the temporal pattern of the uncaging pattern delivered at the individual spine and the relative location of the neighbors.

It is well-established that, depending on the availability of plasticity-related proteins, the 30-Reg pattern induces short- or long-lasting LTP at the stimulated single dendritic spines. We have recently shown that while NSP-Uni induces long-lasting sLTP that is protein synthesis-dependent, NSP-Beg and NSP-End only induce short-lasting sLTP (up to 1 h post-induction; Argunsah and Israely, 2023). Neither this study nor other previous studies (Matsuzaki et al., 2004; Harvey and Svoboda, 2007; Govindarajan et al., 2011) have shown any heterosynaptic effects at the neighbors of the 30-Reg stimulated spines. Here, through further observing these neighbors for a longer time and analyzing them in different spatial loci, we have shown that the neighbors of the 30-Reg stimulated spines not only do not show any structural plasticity over time but also do not exhibit either any location-dependent plasticity differences (Figure 2) or any plasticity gradients at the local neighborhood (Figures 4A, B). The only significant irregularity presented by these neighbors was at the CV trends of the inner neighbors of the 30-Reg stimulated spines (Figures 3C, D). No wonder previous studies have concluded that such heterosynaptic changes could not be induced at the local neighbors around the individually activated dendritic spines using the 30-Reg protocol.

Since similar to 30-Reg, NSP-Uni can induce long-lasting sLTP at the stimulated spines, one could expect similar structural dynamics for the neighbors of NSP-Uni stimulated spines to the neighbors of 30-Reg stimulated ones. This assumption mostly holds except for the CV trends of the inner neighbors of the NSP-Uni stimulated spines do not go back to baseline (Figures 3C, D), and while 30-Reg did not cause any plasticity gradient at the local dendritic neighborhood, NSP-Uni showed a significant inner → outer gradient (Figures 4C, D) compared to their baseline.

Studies of spine motility *in-vivo* and *in-vitro* have shown that spine motility is a function of development (Fischer et al., 1998; Dunaevsky et al., 1999; Lendvai et al., 2000). It is the highest in neonates around the time of synaptogenesis and decreases gradually afterward. These studies concluded that dendritic spines are most motile at a time when they are either not receiving any input or receiving sub-threshold input (Majewska and Sur, 2003). Here, using CV as a proxy for spine motility, we showed that naturalistic patterns maintain the motility of the both inner and outer unstimulated neighbors up to 220 min post-stimulation which the 30-Reg pattern does not (Figures 3C–F).

Why does 30-Reg stimulation induce long-lasting sLTP at the stimulated spine but does not create a plasticity gradient in the local neighborhood while NSP-Uni does? It is conceivable that extremely salient synaptic inputs might have some sort of emergency connotation for the stimulated spines which enforces input specificity. It has previously been shown that spontaneous glutamate release modifies the threshold for plasticity at single dendritic spines through the local regulation of NMDARs to spatially limit the synaptic metaplasticity (Lee et al., 2010), and the plasticity induced by the 30-Reg pattern also lowers the plasticity threshold for further induction (Govindarajan et al., 2011) in an NMDAR-dependent manner. Hence, this form of plasticity could also create meta-plastic states that are highly input specific.

We have previously shown that not all three naturalistic patterns induce long-lasting sLTP. Interestingly, neighbors of these NSP-Beg and NSP-End stimulated spines showed the highest level of location-dependent divergence (Figure 2F, red vs. green). We know that these patterns only induce sLTP up to 1 h, and here, we see that the difference between these neighbors increases drastically after this point (Figure 2F). It is known that if a spine has been previously activated by strong stimuli, further weaker stimuli can create higher-than-expected levels of plasticity (Govindarajan et al., 2011). Since the difference between NSP-Beg and NSP-End is the temporal gradient of the uncaging pulses (NSP-Beg has a higher average inter-pulse frequency at the beginning that goes down over time, NSP-End is the opposite), the high-frequency portion of the NSP-Beg could act as a relatively “stronger” stimuli that would help the forthcoming weaker pulses to make a higher impact.

It has recently been shown that PKC activity is not restricted to the stimulated spine when multiple spines are stimulated using 30-Reg (Colgan et al., 2023), which suggests that PKC could spread some distance from the stimulated loci. Additionally, TrkB has previously been shown to regulate long-distance signaling between activated synapses and the nucleus (Harward et al., 2016; Esvald et al., 2020; Moya-Alvarado and Bronfman, 2020). Although the differences we report here are caused by single spine activation, the temporal structure of the naturalistic patterns might induce a similar non-linear effect which could cause the reported differences at *inner* vs. *outer* neighboring spines through interactions between TrkB and PKC.

Here, we present the effects of regular and naturalistic stimulation patterns on the unstimulated neighbors of stimulated single dendritic spines. Our results suggest that, on average, none of the patterns cause any drastic structural modification at the unstimulated spines. Only by separating the neighbors into two groups depending on whether they are in between the stimulated spine and the main dendrite vs. in between the stimulated spine and the distal end of the local dendrite, divergences emerge. We have seen that outer-neighboring spines are more likely to express structural modifications and the temporal skewness of the uncaging pattern could lead to divergent structural dynamics at these spines. Additionally, we have shown that NSP-Uni, and NSP-Beg to a lesser extent, patterns create plasticity gradients at the neighbors of the stimulated spines. This study presents the first evidence of relative location-dependent structural modifications at the unstimulated neighbors of the individually stimulated spines.

## Data availability statement

The raw data supporting the conclusions of this article will be made available by the authors, without undue reservation.

## Ethics statement

The animal study was approved by Portuguese Veterinary Authority (DGV). The study was conducted in accordance with the local legislation and institutional requirements.

## Author contributions

AA: Conceptualization, Data curation, Formal analysis, Funding acquisition, Investigation, Methodology, Project administration, Resources, Software, Validation, Visualization, Writing—original draft, Writing—review and editing. II: Conceptualization, Funding acquisition, Project administration, Resources, Supervision, Writing—review and editing.

## Footnotes

^1^ https://doi.org/10.5281/zenodo.7871541
